# Turtle Shell Kinesis Underscores Constraints and Opportunities in the Evolution of the Vertebrate Musculoskeletal System

**DOI:** 10.1093/iob/obad033

**Published:** 2023-09-04

**Authors:** G A Cordero

**Affiliations:** Department of Animal Biology, Centre for Ecology, Evolution and Environmental Changes, University of Lisbon, 1740-016 Lisbon, Portugal

## Abstract

Species groups that feature traits with a low number of potentially variable (evolvable) character states are more likely to repeatedly evolve similar phenotypes, that is, convergence. To evaluate this phenomenon, this present paper addresses anatomical alterations in turtles that convergently evolved shell kinesis, for example, the movement of shell bones to better shield the head and extremities. Kinesis constitutes a major departure from the evolutionarily conserved shell of modern turtles, yet it has arisen independently at least 8 times. The hallmark signature of kinesis is the presence of shell bone articulations or “hinges,” which arise via similar skeletal remodeling processes in species that do not share a recent common ancestor. Still, the internal biomechanical components that power kinesis may differ in such distantly related species. Complex diarthrodial joints and modified muscle connections expand the functional boundaries of the limb girdles and neck in a lineage-specific manner. Some lineages even exhibit mobility of thoracic and sacral vertebrae to facilitate shell closure. Depending on historical contingency and structural correlation, a myriad of anatomical alterations has yielded similar functional outcomes, that is, many-to-one mapping, during the convergent evolution of shell kinesis. The various iterations of this intricate phenotype illustrate the potential for the vertebrate musculoskeletal system to undergo evolutionary change, even when constraints are imposed by the development and structural complexity of a shelled body plan. Based on observations in turtles and comparisons to other vertebrates, a hypothetical framework that implicates functional interactions in the origination of novel musculoskeletal traits is presented.

## Abstract (es)

Resumen

Los grupos de especies que presentan rasgos con un bajo número de estados de carácter potencialmente variables (evolutivos) tienen más probabilidades de evolucionar repetidamente fenotipos similares. El presente artículo aborda las alteraciones anatómicas en las tortugas que evolucionaron de manera convergente el cierre cinético del caparazón y plastrón. El cierre cinético mejora la protección de la cabeza y las extremidades. Este rasgo constituye una desviación importante en la historia evolutiva de las tortugas modernas, sin embargo, ha surgido independientemente al menos ocho veces. La cinesis se define por la presencia de articulaciones óseas o “bisagras” que surgen a través de procesos similares de remodelación esquelética en especies que no comparten un ancestro común reciente. No obstante, los componentes biomecánicos internos relacionados con la cinesis pueden diferir en especies tan distantemente relacionadas. Las articulaciones diartródias complejas y las conexiones musculares modificadas expanden los límites funcionales del cuello y las cinturas escapulohumeral y pélvica de una manera específica del linaje. Algunos linajes incluso exhiben movilidad de vertebras torácicas y sacras para facilitar el cierre cinético. Dependiendo de la contingencia histórica y la correlación estructural del sistema musculoesquelético, varias alteraciones anatómicas han producido resultados funcionales similares durante la evolución convergente de la cinesis. Las diversas iteraciones de este fenotipo ilustran el potencial del sistema musculoesquelético de los vertebrados para experimentar cambios evolutivos, incluso cuando existen limitaciones impuestas por el desarrollo y la complejidad estructural del plan corporal de las tortugas. Sobre la base de observaciones en tortugas y comparaciones con otros vertebrados, se presenta un marco hipotético que implica interacciones funcionales en el origen de nuevos rasgos musculoesqueléticos.

## Introduction

Convergent evolution generally defines phylogenetic patterns wherein similar phenotypes are observed in species that do not share a recent common ancestor, which is often attributed to common selective pressures in distantly related lineages (Wake et al. 2011; Stayton 2015; Mahler et al. 2017). Furthermore, character evolution models predicted a higher probability of convergence in species groups (clades) that feature somewhat invariable morphological traits (Bock 1963; Donoghue and Ree 2000). Although evolution generally proceeds via the rearrangement of pre-existing morphologies, not all organismal structures are free to vary (Bock 1959; Frazzetta 1976; Wainwright 2007). In other words, converging upon the same adaptive strategy is increasingly probable when there are inherently fewer options for a trait to undergo change, that is, constraints. The definition of constraints on phenotypic evolution is often debated (Maynard Smith et al. 1985), though herein it mainly concerns tissue formation (developmental) processes that influence the extent to which functionally relevant structures vary during macroevolution, that is, how major interspecific changes in anatomy or morphology take place across vast timescales (Galis et al. 2018).

The potential for traits to evolve (evolvability) might be constrained by the inherited ancestral state of a clade (Bock 1963; Donoghue and Ree 2000; Losos 2011). Evolution may therefore follow predictable trajectories within the bounds of an ancestral architecture, though divergence is feasible while retaining key ancestral features in all lineages of a clade (Losos 2011). An example of anatomical constancy during adaptive phenotypic diversification concerns the turtle's shell. Modern turtles (Testudines) feature 10 thoracic ribs, of which 8 become tightly bound to each other via extensions of ossified (calcified) tissue that comprise the bones of the dorsal shell (carapace) (Zangerl 1969; Lyson and Bever 2020). Similarly, the ventral shell (plastron) is a composite of modified skeletal elements that are hypothesized to be homologous to the clavicle, sternum, and gastralia (floating ribs) (Rice et al. 2016). This ancestral shelled configuration has been maintained over the last 210 years of evolution (Lyson and Bever 2020), despite repeated specialization to freshwater, marine, and terrestrial environments (Cordero 2017).

**Fig. 1:**
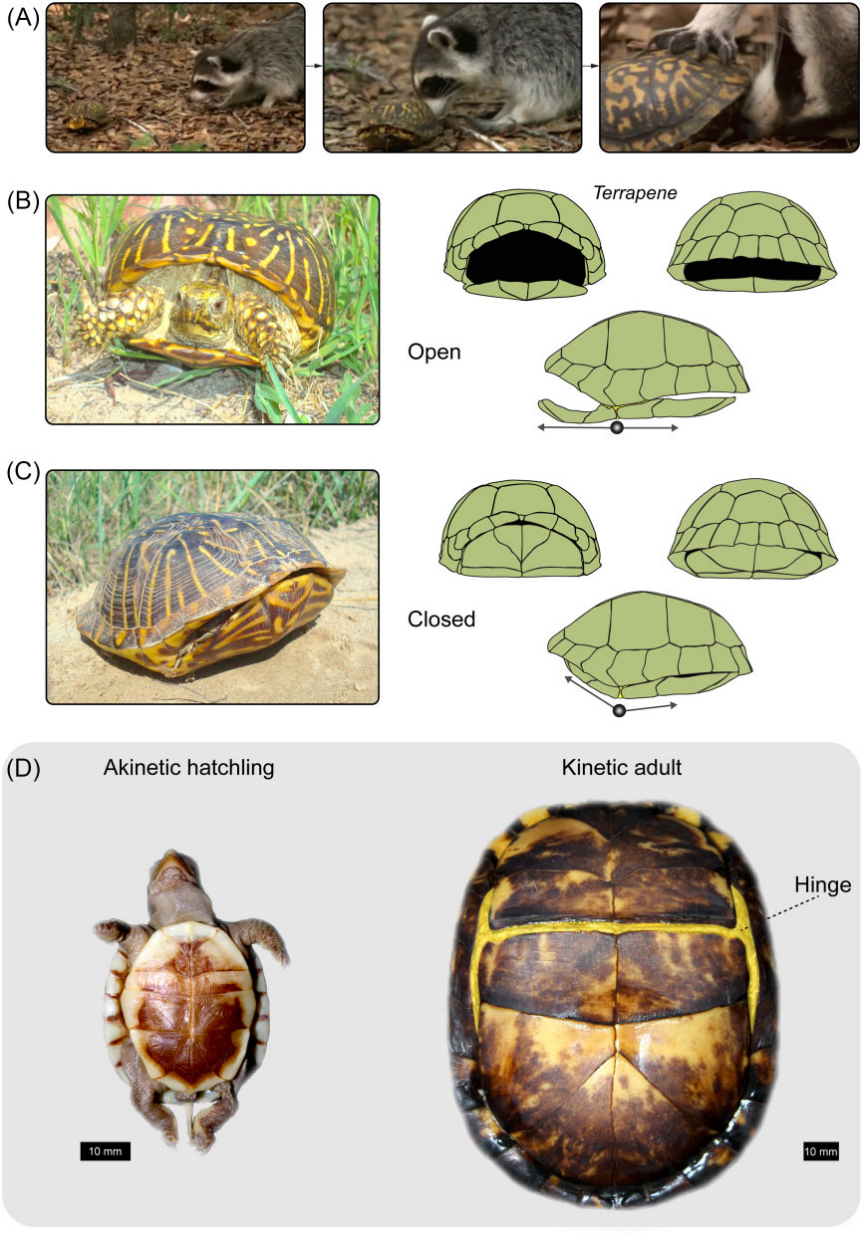
(**A**) Anti-predatory defense is a potential adaptive function of shell kinesis in some turtle species. Shown is an eastern box turtle (*Terrapene carolina*) engaging in shell closure after encountering a potential predator (images modified from *Life in Cold Blood* with permission from the British Broadcasting Company). (**B**) An ornate box turtle (*Terrapene ornata*) in an exposed (open) stance. (**B**—right panels) Anterior, posterior, and lateral views of lowered plastral lobes are shown. (**C**) In the defensive (closed) stance, the limbs, head, and tail are withdrawn while remaining shielded by the elevation of the plastral lobes via a kinetic hinge joint. (**D**—left panel) The plastron lacks the said hinge and is thus akinetic in hatchling *T. carolina*. (**D**—right panel) The hinge is otherwise a conspicuous feature (highlighted for clarity) of the adult plastron of *T. carolina*.

The encasement of the limb girdles within the turtle's shell, along with a heavy and rigid thoracic region, renders improbable the evolution of climbing agility, high-speed sprinting, flight, gliding, and undulation-based swimming. The range of evolvable musculoskeletal phenotypes and functions that is otherwise observed in other vertebrate clades is reduced in turtles. Even so, the development of thoracic rigidity has likely channeled turtle evolution to a phenotypic space that is unavailable to other vertebrate clades, yielding new opportunities by which the musculoskeletal system may be modified. This paper addresses such opportunities, as well as related constraints, by examining the convergent evolution of shell kinesis, that is, the ability to actively move shell bones such that the head and extremities are better concealed (Fig. 1) (Bramble 1974; Bramble and Hutchison 1981; Bramble et al. 1984). Musculoskeletal alterations will be reviewed to discuss how architectural challenges were mitigated in evolution: The head and limbs must be retracted deeply into a rigid body cavity that also houses the limb girdles and vital organs, including lungs that do not expand in conjunction with the thoracic region during inhalation, as in other vertebrates (Landberg et al. 2003).

Some of the muscle connections that power kinesis were probably absent from the earliest fully shelled turtles within Testudinata. The repeated contraction of muscles with connections that are novel, relative to the expected ancestral condition, is hypothesized to contribute to the structural reorganization of bone sutures and delayed emergence of kinetic hinges in juveniles (Cordero et al. 2018b). The broader evolutionary relevance of this form of developmental plasticity will be discussed. This paper also attempts to address the assumption that distinct musculoskeletal rearrangements in embryos eventually lead to similar end products of development in adults: a kinetic hinge joint on the shell (Bramble 1974; Bramble and Hutchison 1981; Bramble et al. 1984). Indeed, the convergence of shell kinesis may well conceptualize a “many-to-one mapping” scenario in the evolution of form-to-function relationships of the musculoskeletal system (Wainwright 2007). Toward this goal, this paper sets out to link phylogenetic patterns with ontogenetic processes to ascertain the extent to which species that do not share a recent common ancestor arrived at analogous functional outcomes via similar or dissimilar phenotypes. Through the comparative approach employed herein, phenotypes that are either rare or unprecedented in the evolution of the turtle musculoskeletal system are assumed to reflect constraints on evolution, as well as insufficient selective pressure for a particular evolutionary change to occur. The former and the latter are discussed for select turtle taxa with the most specialized forms of shell kinesis, while inferring developmental or structural constraints based on a review of the best presently available comparative embryological, anatomical, and functional data.

## Multiple evolutionary origins of shell kinesis

**Fig. 2:**
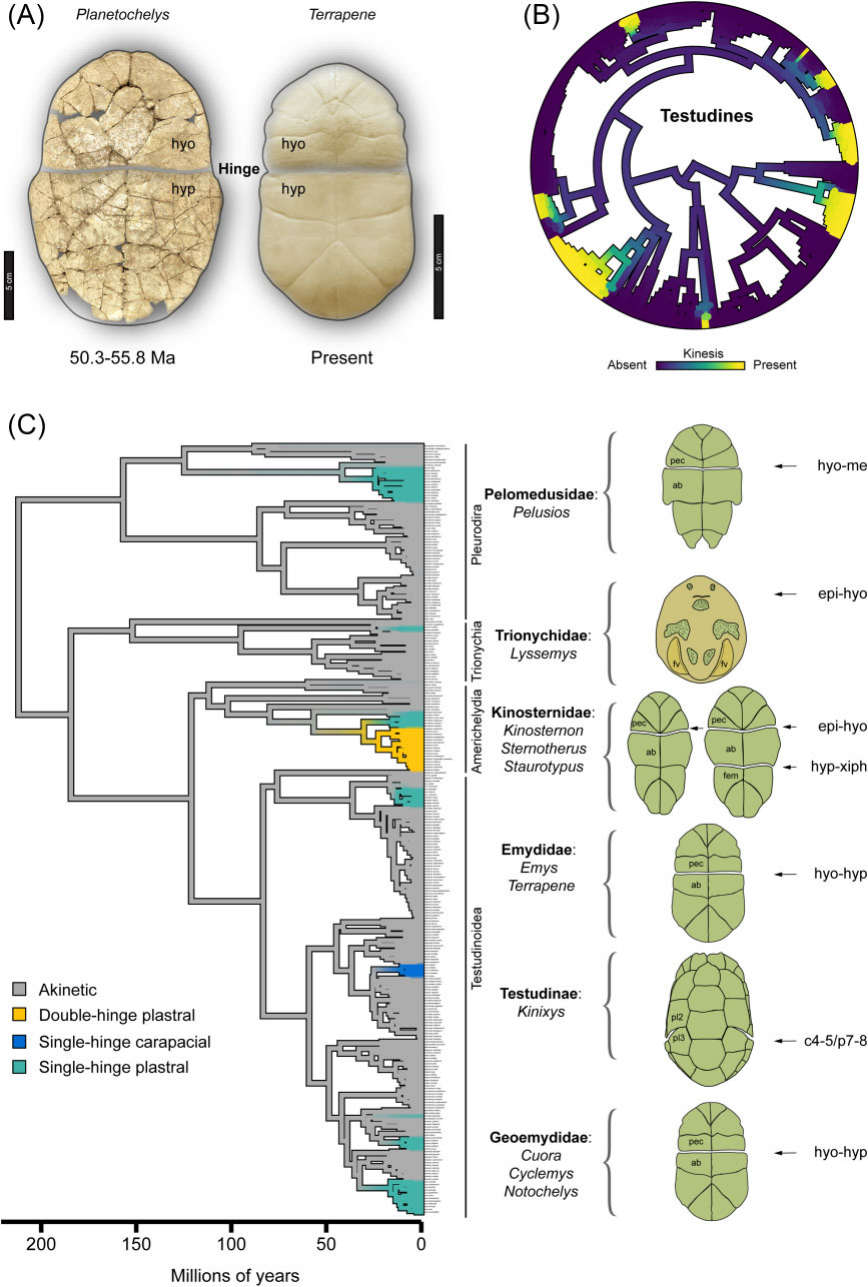
(**A—**left) A hinge between the hyoplastron (hyo) and hypoplastron (hyp) is evident in several extinct turtles dated to the early Paleogene, for example, *Planetochelys dithyros* (University of California Museum of Paleontology 120000 holotype; image copyright: David P. Strauss CC-BY 3.0). *Planetochelys* resembles extant kinetic-shelled species (**A**—right: Skeletonized plastron of *T. carolina*). (**B**) Shell kinesis evolved at least eight times in extant turtles, as mapped on the phylogeny of Thomson et al. (2021). (**C**) Ancestral state reconstruction of the different shell kinesis phenotypes. The color gradients in panels **B**–**C** summarize 100 stochastic character map ancestral state simulations generated by the *Phytools* R package (Revell 2012). *Pelusios* is the only sideneck turtle (Pleurodira) with shell kinesis, see hinge on the hyo and mesoplastron (me) and on sulci of the pectoral (pec) and abdominal (ab) scutes. Plastral kinesis in softshell turtles (*Lyssemys* spp.) is evident by a flexible articulation on the epiplastron (epi) and hyo bones, which is discerned by transverse skin creases. Kinosternids feature either double or single plastral kinesis with kinetic hinges on the epi–hyo or hyp–xiphiplastron (xiph) that match the sulci of the pec-abdominal (ab) and femoral (fem) scutes. The tortoise genus *Kinixys* is the only taxon with a carapacial hinge, which is situated at costal (c) bones 4–5 and peripheral bones 7–8 and corresponds to pleural (pl) scutes 2–3 and marginal scutes 7–8 (not labeled).

Shell kinesis has evolved repeatedly across different branches of the turtle tree of life, as evidenced by highly fibrous articulations (hinges) that permit considerable movement (kinesis) of shell bones (Figs. 1 and 2). This might be adaptive because it reduces the soft tissue area that is exposed to the external environment and potential predators (Minckley 1966; Green 1988; Wygoda and Chmura 1990; Murphy et al. 2016; Preston et al. 2020) (Fig. 1). It may have also secondarily evolved to accommodate the wide gape of megacephalous species (Bramble et al. 1984), while some species may feature transient female-specific kinesis that is presumably related to egg laying (Pritchard 2008). Kinesis wherein both female and male adults predictably develop a kinetic hinge will be reviewed. This discussion assumes a pattern-based definition of convergent evolution that does not presuppose a shared selective pressure among distantly related lineages (Stayton 2015), but rather focuses on the frequency, variants, and earliest known evolutionary origins.

### Historical trends in the fossil record

The fossil record is indispensable to the study of evolvability and the deep evolutionary conservation of traits as a result of developmental constraints (Love et al. 2021). Turtles have among the best fossil record in vertebrate animals, which aids in clarifying the spectrum of shell phenotypes that may be expressed. One or two transverse lines etched across the plastron of fossils are often interpreted as kinetic hinges, owing to the frequent spatial congruence of scute boundaries (sulci) and bone sutures that is observed in living taxa (Fig. 2A). As such, a rudimentary form of shell kinesis possibly arose during the Santonian–Campanian stages of the Cretaceous, ca. 86.3–70.6 Ma (reviewed by Sukhanov 2000). The sulci of gular and pectoral scutes seemingly align with the sutures of epiplastron bones, while the pectoral scute sulci align with the anterior entoplastron and hyoplastron sutures of *Shachemys baibolatica* (Sukhanov 2000). Nonetheless, further evidence is needed to explicitly test the hypothesis that *Shachemys* is the earliest kinetic-shelled turtle.

The best supported inference is that forms of shell kinesis that resemble the modern condition most likely originated in the Paleogene. Several extinct taxa from the early Paleogene clearly featured characters that are structurally analogous to those of extant species (Hutchison 2013; Tong et al. 2016; Joyce and Claude 2020). The well-preserved fossil material of *Planetochelys* spp. bears a striking resemblance to extant kinetic-shelled *Terrapene* and *Cuora* of Testudinoidea (Fig. 2A–C). Characters unrelated to kinesis, however, do not support that *Planetochelys* was ancestral to modern Testudinoidea (Hutchison 2013), as it was tentatively placed within Kinosternoidea (Joyce and Bourque 2016). A hyoplastral–hypoplastral hinge was described in another member of Kinosternoidea from the early Paleogene: *Cardichelyon rogerwoodi* (Joyce and Claude 2020). The first testudinoid that featured kinesis is *Anhuichelys* spp., which is presently recognized as a stem member of the Testudinidae tortoise family (Tong et al. 2016). Fossils indicate that *Anhuichelys* featured a single anterior epiplastral–entoplastral/hyoplastral hinge, but may have also concurrently developed a hypoplastral–xiphiplastral hinge (Tong et al. 2016). Some softshell turtles might have featured kinetic shells during the early Paleogene, as suggested by the putative entoplastral–hyoplastral hinge of *Hutchemys remendium* (Joyce et al. 2009).

Shell kinesis arose independently in ancient subclades of Kinosternoidea, Testudinoidea, Trionychia, and possibly Adocusia (e.g., *Shachemys*). Future studies may benefit from integrating data on extinct and extant lineages and may reveal additional convergent origins of shell kinesis. Fossils demonstrate that all plastral bone sutures have the potential to be transformed into kinetic joints. No extinct taxon with a carapacial hinge has been described, suggesting the non-exclusive possibilities that the carapace is not as structurally amenable to the formation of kinetic joints, weak selection for carapacial kinesis, or that the presence of a carapacial hinge is more challenging to discern in fossils. Altogether, the fossil record strengthens the historical context for hypotheses on the range of structural shell rearrangements that are feasible in evolution.

### Convergence in living species: multiple means to achieve similar functional outcomes

**Fig. 3:**
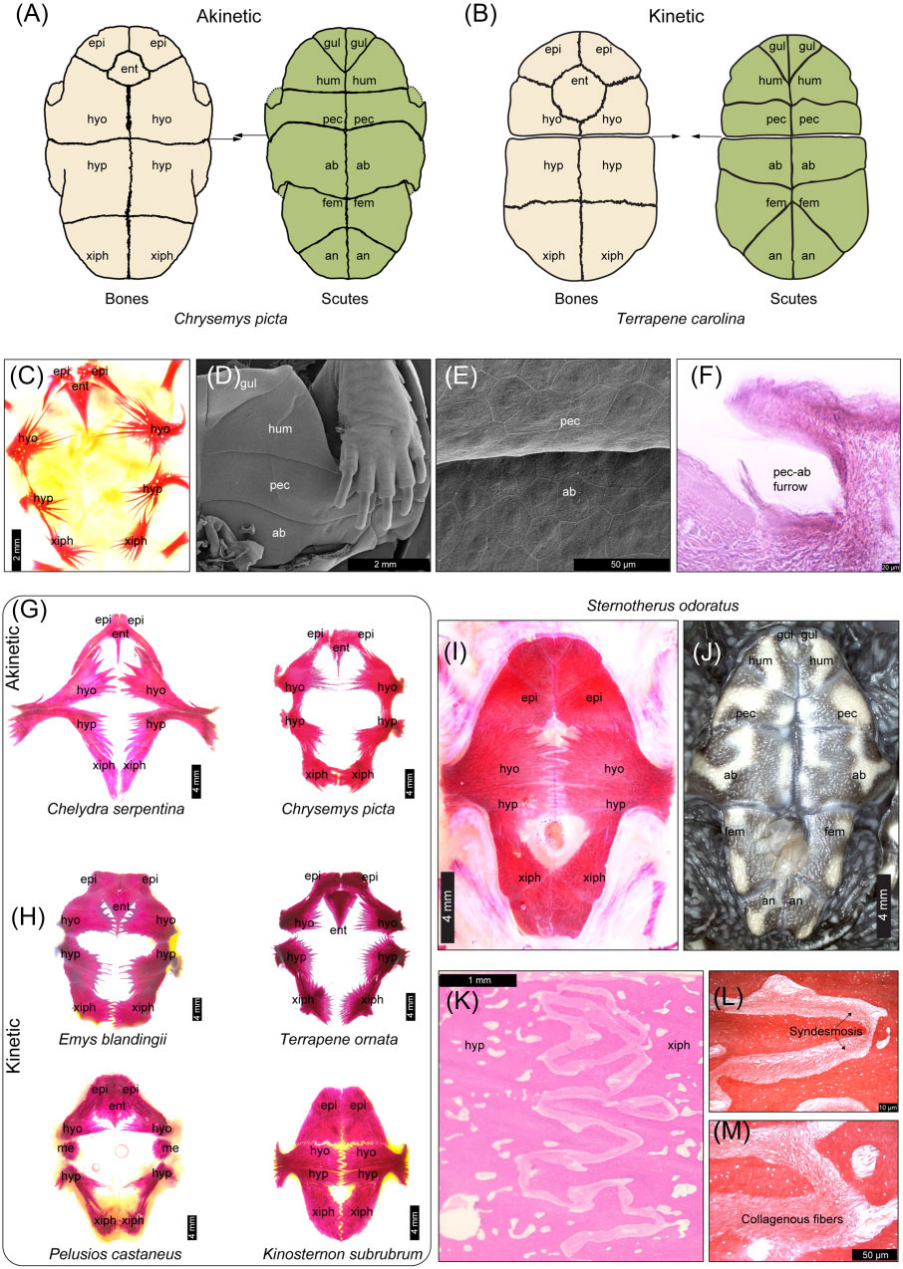
(**A**) Bone sutures are generally not aligned with scute sulci. (**B**) In kinetic-shelled turtles, the hinge coincides with sulci and related sutures. (**C**) Shell development is incomplete in embryos; see plastron ossification centers (in alizarin red) in stage-21 *Chrysemys picta*. (**D–E**) By stage 21, the plastron is covered by keratinous scutes (panel **E**: Keratinocytes are visualized with scanning electron microscopy) with well demarcated boundaries (sulci). (**F**) In hatchlings, the pectoral (pec)-abdominal (ab) scute sulci (*C. picta* in a longitudinal hematoxylin and eosin [H&E] section) exhibit a furrow-like morphology. (**G**) In hatchlings of akinetic-shelled species, early sutural contact (interdigitation) can be observed. (**H**) Kinetic-shelled species display interdigitation, except at the hyoplastron (hyo)-hypoplastron (hyp) in *Terrapene ornata* or hyo-mesoplastron (me) in *Pelusios castaneus*. The epiplastron (epi) and entoplastron (ent) exhibit early sutural formation in all sampled hatchlings. All bones, including the xiphiplastron (xiph), display sutural contact in *K. subrubrum* (**H**—bottom right). (**I–J**) The plastron is also highly ossified in other hatchling kinosternids (*Sternotherus odoratus*; fem = femoral; an = anal). (**K**) A longitudinal H&E section of fully jointed (syndesmotic) hyo–hyp bones in *C. picta*. (**L–M**) Mature sutures feature collagenous connective tissue (longitudinal section of *Glyptemys insculpta* stained in Verhoeff-Van Gieson solution).

Unlike in fossils, the fibrous connective tissue of a *bona fide* (moveable) hinge joint can be indisputably identified by determining the congruence of scute sulci and bone sutures (Fig. 3A–F). *In vivo* and *ex vivo* shell bone movement aid in ascertaining whether putative hinge joints are kinetic, though this is sometimes nuanced in species that feature moderate structural flexibility without modified sutures. Underdeveloped forms of kinesis suggest that the hinge and its related characters could be vestigial or have lost adaptive value in some species, even if shell movement may be potentially induced by cervical and limb girdle muscles (Bramble 1974). Based on the presence of such correlated modifications, at least eight independent gains of kinesis occurred in evolution (Cordero et al. 2018b). Still, there is some uncertainty within some clades, such as in emydid box turtles (*Terrapene* + *Emys*). It was initially proposed that the last shared common ancestor of *Terrapene* and *Emys* featured kinesis (Bramble 1974), though this hypothesis was not robustly supported by ancestral state reconstruction analyses (Feldman and Parham 2002; Angielczyk et al. 2011; Cordero et al. 2018a). However, because *Terrapene* and *Emys* were recovered as sister lineages in the most recent phylogeny of Thomson et al. (2021), a single origin of plastral kinesis within Emydidae cannot be entirely ruled out (Fig. 2C).

Assuming the topology of Thomson et al. (2021), two independent origins of kinesis (*Cyclemys* and *Cuora*) are well substantiated in Geoemydidae (Fig. 2B and C). The geoemydid *Notochelys platynota* might also feature a hyoplastron–hypoplastron hinge, though this was not conclusively confirmed by the analyses of Bramble (1974). If kinesis is confirmed in *N. platynota*, then three independent origins of plastral kinesis in Geoemydidae may be inferred (Fig. 2B and C). In Testudinoidea, *Kinixys* (African hinge-back tortoises) is the only taxon with carapacial kinesis (Fig. 2C), which is surprising because it might be expected that selection for shell closure would favor the relatively fewer complex changes associated with the development of plastral kinesis. Species may feature up to two plastral hinges in Kinosternidae (Bramble et al. 1984) (Fig. 2C). In softshell turtles, *Lyssemys* features an unusual epiplastron–hyoplastron hinged plastron with skin flaps (valves) that aid in concealing the limbs and head during retraction (Fig. 2C). Lastly, *Pelusios* is the only sideneck taxon (Pleurodira) that features plastral kinesis (Bramble and Hutchison 1981) (Fig. 2C). That the location of hinges is variable is an important observation because it suggests that different suites of correlated musculoskeletal traits may have evolved in accordance with the shell architecture of a given subclade, though other factors may also be important, as discussed below.

## Convergence but not entirely via similar ontogenetic processes

Turtle shell bones are not entirely different from those of other vertebrates. In fact, dermal bones and sutures of the human skull share similar microstructural features with the turtle's plastron (Ishikawa et al. 2019). However, in turtles, the rather slow pace of dermal bone maturation provides an opportunity for bone sutures, which typically fuse tightly with one another, to be repatterned into moveable hinge joints. What makes this transformation possible? In the following sections, a model for shell hinge differentiation is presented while discussing similarity, or lack thereof, in the multifaceted changes that precede the delayed emergence of such unusual skeletal articulations.

### Constraints counteracted by skeletal plasticity: the delayed emergence of “hinge” joints

**Fig. 4:**
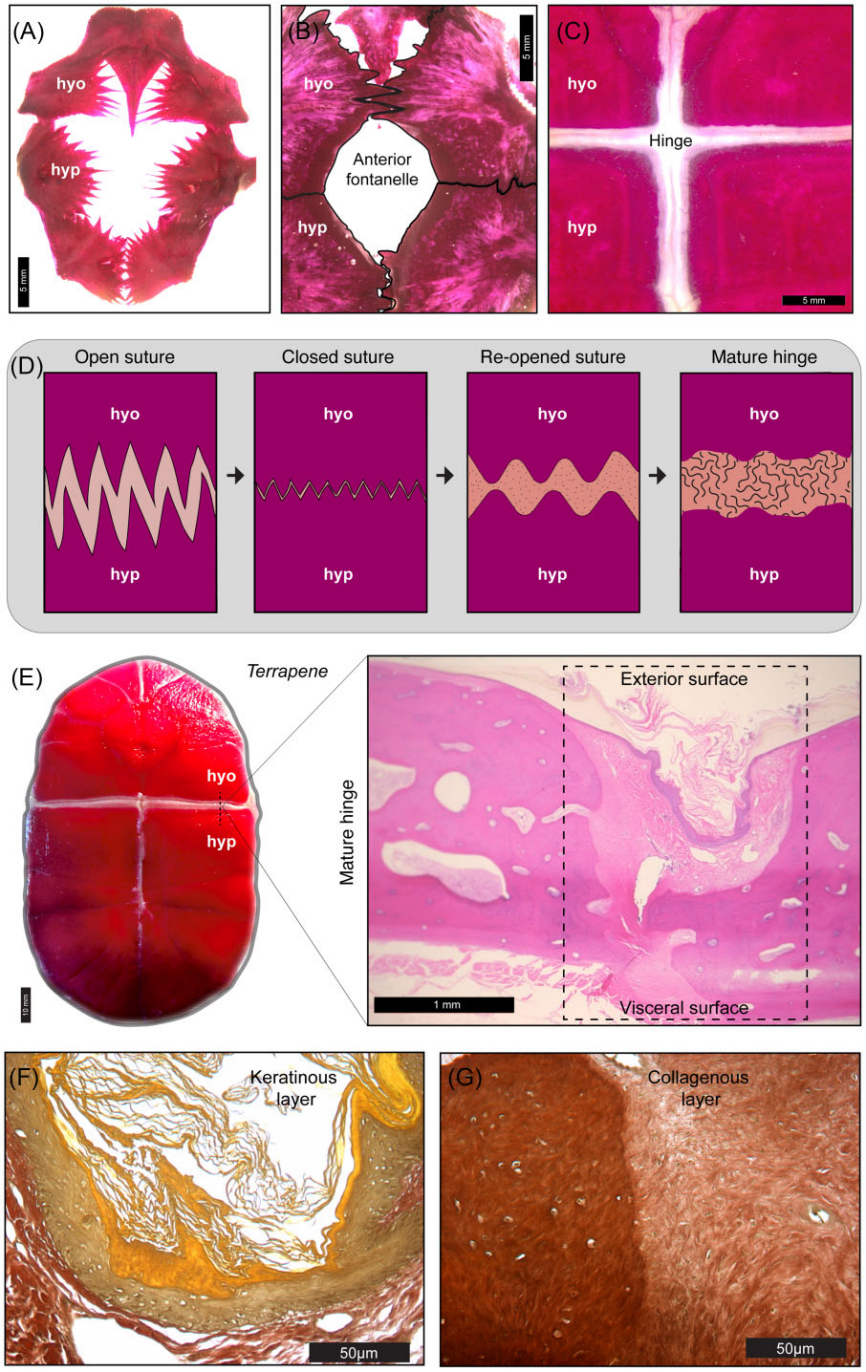
(**A**) At 1 year after hatching, the ossification centers (stained in alizarin red) of the hyoplastron (hyo) and hypoplatron (hyp) exhibit partial contact in *Terrapene ornata*. (**B**) At 3 years after hatching, the hyo–hyp sutural junction spans nearly the entire width of bones, while midline sutural contact remains incomplete (see fontanelle). (**C**) In adults, the anterior fontanelle closes, though the hyo–hyp suture sutural junction is separated by thick connective tissue. (**D**) A model of sutural repatterning is presented, with post-hatching age increasing from left to right. The hyo–hyp suture is expected to close and subsequently re-open during the skeletal remodeling process that gives rise to a mature hinge joint. (**E**) An alizarin red-stained adult *Terrapene carolina* plastron (left panel) displays the external surface of the hyo—hyp hinge. (**E—**inset) The gross anatomy of the mature hinge is shown in a longitudinal section stained in hematoxylin and eosin. (**F–G**) High-magnification views of corneous (left—keratinous layer) and fibrous (collagenous layer) hinge tissue in longitudinal sections stained in Verhoeff-Van Gieson solution.

A bewildering aspect of the convergent evolution of shell kinesis is that no external signs of a kinetic hinge are apparent after the end of embryonic development (Legler 1960; Richmond 1964; Cordero et al. 2018b, 2022; Cordero 2021) (Figs. 1D and 3G–J). It was once hypothesized that a lack of sutural contact eventually facilitates hinge differentiation via the mere expansion of fibrous connective tissue (Pritchard 2003), which is typically minimal in normal shell sutures (see "syndesmosis" in Fig. 3K–M). Scute sulci and sutures would then need to achieve spatial juxtaposition during ontogeny (Bramble 1974; Ernst et al. 1997; Pritchard 2003). In agreement with this idea, shell elements are spatially rearranged during post-hatching growth (Fig. 4). Still, sutures initially follow a normal pattern of development before a hinge arises via secondary skeletal remodeling (Cordero et al. 2018b, 2023). This transformation underscores that the plastic properties of skeletal tissue (Lanyon and Rubin 1985; Currey 2002; Cowin 2004; Franz-Odendaal 2011; Herring 2011), which are expected to be shared by all vertebrate animals, may counteract constraints and thus promote the evolution of novel phenotypes (Wagner 2014).

In the context of skeletal development, plasticity refers to the actual malleability of the chondral and osseous materials that comprise bone tissue. This is a key distinction because skeletal plasticity, beyond cases wherein it may be triggered by the external environment (Wikelski and Thom 2000; Lázaro et al. 2017), should not be considered the sole determinant for the expression of a skeletal trait. Instead, skeletal plasticity describes reaction norms that typically involve mechanical inputs that originate within the internal environment of the organism, for example, muscle-derived forces (Newman and Müller 2001; [Bibr bib42][Bibr bib42]). In diverse vertebrate animals, it has been hypothesized that the activation of such reaction norms is most likely promoted by selection for the proper muscle attachment, size, and orientation, which are presumably correlated traits that are heritable and originate in early embryonic development (Botelho et al. 2015; Hu and Albertson 2017). There is a growing body of experimental evidence in line with the assumption that skeletal reaction norms are a means by which evolution may proceed along the lines of least resistance (Young and Badyaev 2007). Otherwise, natural selection for changes in skeletal phenotypes may translate to changes in morphogenetic gradients or highly intertwined inductive tissue interactions that are expected to be highly invariant because any deviation would result in lethality via malformation of the primordial skeleton (Galis et al. 2018; Galis and Metz 2019). Consequently, evo-devo theoretical models have predicted that selection for highly specialized skeletal traits favors changes in the late stages of embryonic development, that is, after the ancestral skeleton is established along with correlated muscle connections (Alberch 1985; Alberch and Blanco 1996). Turtles seem to adhere to this trend, though additional structural constraints might be imposed by a shelled body that encapsulates the shoulder and pelvic girdles. The suturing of shell bones on its own is not necessarily a constraint, as suturing may concurrently enhance the protective capacity of the shell during interactions with predators. Indeed, some miniaturized kinosternid turtles exhibit accelerated shell bone suturing shortly after hatching (Cordero 2021).

Interdigitation of bony processes and suture closure does occur at the contact site of adjacent bones in juveniles, before external proliferation of hinge connective tissue is apparent (Fig. 4A–C). Following the closure of a transient suture (Fig. 4D), skeletal remodeling widens the space between bones (Fig. 4E). This gap is occupied by dense collagenous fibers with a corneous (keratinous) external surface (Fig. 4E–G). In addition, the point of contact between the carapace and plastron, that is, the shell bridge, undergoes resorption and gains flexibility by the deposition of connective tissue in emydid and geoemydid species with plastral kinesis (Cordero et al. 2018b). The timing of suture closure, reopening, and remodeling may vary. Notably, early plastron bone suturing is already evident by the end of embryonic development in kinetic-shelled kinosternids (Cordero 2021). Hutchinson and Bramble (1981) hypothesized that the abdominal scutes were subdivided during hinge development. Because the hatchling scute formula matches the adult condition in kinosternids (see Fig. 3H–J and Legler and Vogt 2013), the hypothesis of scute subdivision is refuted and the standard nomenclature for plastron scutes applies to Kinosternidae (see Cordero and Vlachos 2021). In any case, repatterning of keratinous tissue most likely follows the re-opening of sutures in Kinosternidae and other taxa. Sutural re-opening began before any external signs of scute sulci degradation could be observed in African hinge-back tortoises (Cordero et al. 2023). By contrast, plastron sutural degradation has also been observed but without alignment of corresponding scute sulci in species with female-specific kinesis (*Heosemys spinosa*), which yielded an incompletely differentiated hinge (Mertens 1942). Similar observations in other species motivated the hypothesis that hormonally driven bone resorption may also contribute to hinge differentiation (Legler and Vogt 2013). This alternative hypothesis has not yet been experimentally assessed.

**Fig. 5:**
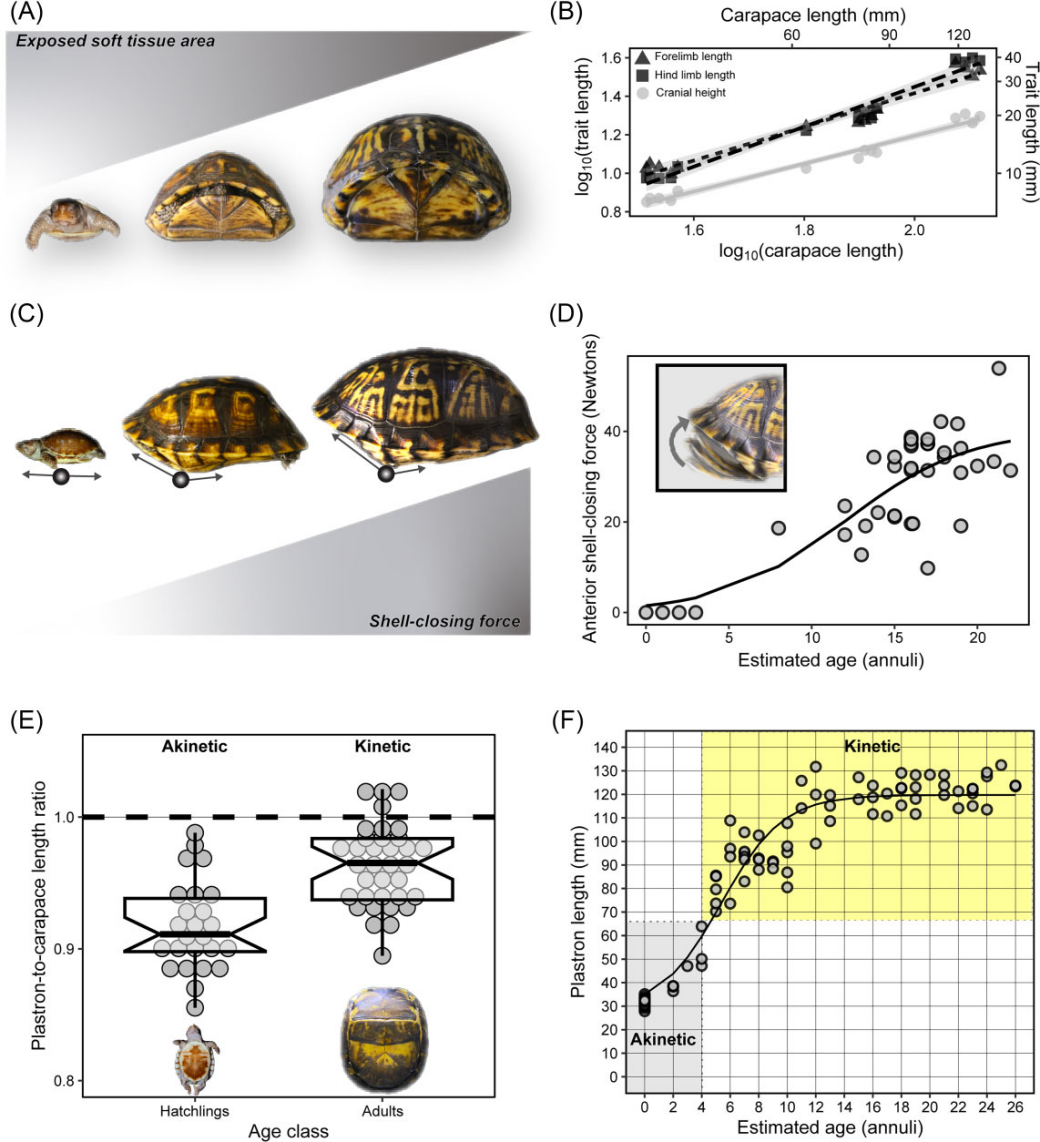
(**A**) The total exposed soft tissue area should decrease as *Terrapene carolina* grows because the plastral lobes can be raised and internal space to withdraw the limbs and head increases. (**B**) Relative to body size (represented by carapace length), cranial height (crh: grey circles) tends to increase at a slower rate (relative to carapace length) than the limbs; see log–log fitted regression lines. (**C**) Shell-closing capacity is facilitated by the degree of hinge dorsoventral flexion in juveniles. (**D**) Anterior shell-closing force increases as a function of age. A non-linear model was fitted to shell-closing data reported by Preston et al. (2020) for individuals >5 years post-hatching, together with expected values for akinetic individuals < 4 years post-hatching that cannot generate measurable shell-closing force. Akinesis was inferred in specimens from Cordero et al. (2019). (**E**) The plastron-to-carapace length ratio approaches a one-to-one relationship once kinesis is achieved in adults (notches represent 95% confidence intervals). (**F**) A logistic growth curve with the expected plastron length and age of akinetic juveniles (grey) and mature kinetic individuals (yellow). Plastron length and age (plastron annuli) data are from Cordero et al. (2019).

Prior to repatterning in emydid box turtles, the transiently closed hyoplastron–hypoplastron suture may exhibit limited mobility during head and neck retraction in *Terrapene carolina*. Dorsoventral flexion of the incipient hyoplastron–hypoplastron, in conjunction with tissue repatterning, gradually increases the extent to which the plastral lobes are elevated while shielding soft tissue regions (Cordero et al. 2019) (Fig. 5A). The head grows slower relative to the shell and is thus able to be withdrawn deeper into the body cavity as dorsoventral flexion of the plastral lobes is achieved in juveniles (Fig. 5B and C). The contractile forces generated by the repeated adduction of the limb and neck retractor musculature are expected to intensify as muscles grow. In vertebrates, muscle force scales positively with increasing body size during ontogeny (e.g., bite force in bats; Stanchak et al. 2023). In box turtles, the occlusion of the plastron and carapace produces a “pinch” force that increases with body size (see Preston et al. 2020; Xiao et al. 2022) (Fig. 5D). Occlusion is enhanced by a nearly one-to-one relationship between the lengths of the plastron and carapace in adults (Fig. 5E).

Hinge differentiation, as depicted in Fig. 4D, is hypothesized to be a response to changes in the mechanical stress regime experienced by developing shell tissue (Cordero et al. 2018b; Cordero et al. 2019) (Fig. 5D). The hinge may be considered an irreversibly plastic trait (within the lifetime of an organism) whose expression is activated if the appropriate tissue structural parameters (thresholds) are met in ontogeny, that is, age, shell dimensions, muscle-derived forces, and size of head and limbs (Fig. 5A–F). It is surmised that the structural conditions that stimulate hinge maturation are met at approximately 4 years post-hatching, when body size increases rapidly and space becomes available within the body cavity to optimally retract and conceal the head and limbs (Fig. 5C). Body size is a phenotypic parameter, or latent liability, that determines the expression of threshold traits (Reid and Acker 2022). A latent liability describes a heritable quantitative trait that, when reaching a certain threshold value, may determine the expression of another discrete trait via developmental plasticity (Reid and Acker 2022). Although experimental and quantitative genetic evidence are yet unavailable, the comparative anatomical and ontogenetic data discussed herein support a hypothetical model wherein selection for kinesis promotes the optimal skeletal trait dimensions and correlated muscle modifications, that is, latent liabilities, that induce the expression of shell hinge joints at a given body size or muscle force threshold.

Based on the universal properties of skeletal tissue in vertebrate animals, all skeletal articulations have the potential to be transformed into complex joints whose movement may be actively controlled by the organism (Hall 2015; Martin et al. 2015). This potential is only realized if the correlated muscle structures (see section “Diverse muscle ‘reconnections’ underlie functional coupling in the evolution of kinesis”) that eventually control the function of joints are in place and able to provide mechanical cues that cells use to orchestrate the structural reorganization of skeletal tissue during ontogeny (Newman and Müller 2001). This function-induced developmental process has been experimentally validated in laboratory animal models (Müller 1990; Franz-Odendaal 2011; Herring 2011; Zhou 2021), though it is rarely studied within a comparative macroevolutionary context as in the convergent evolution of shell kinesis. Comparative phylogenetic studies have already modeled how extrinsic mechanical forces are distributed throughout the shell (Polly et al. 2016), while measurements of intrinsic forces have recently begun to be studied *in vivo* (Preston et al. 2020; Xiao et al. 2022). Additional studies that specifically model (*in vivo* or *in silico*) ontogenetic shell tissue transformations may further clarify how developmental plasticity facilitated the convergent evolution of shell kinesis.

### How evolutionarily conserved is embryonic development?

The repeated and delayed addition of a hinge to the ontogenetic sequence of distantly related lineages would imply that changes in early development are somewhat biased by an ancestral state configuration. Even so, some integral features of kinetic shell systems likely emerge in embryos, possibly in response to selection for correlated musculoskeletal traits that subsequently enable or promote the mobility of mature shell components in adults (see sections “Diverse muscle ‘reconnections’ underlie functional coupling in the evolution of kinesis” and “Correlated limb girdle alterations, innovation, and functional enhancement”). For instance, the reduced ossification of shell bridge bones in hatchlings foreshadowed the adult condition in emydid box turtles, indicating that this functionally relevant difference is pre-patterned in embryos (Cordero et al. 2018b). Pre-patterning likely involves regional changes in the early proliferation and arrangement of osteoblasts, which, in turn, determine the size and shape of the shell bridge region in hatchlings. Beyond such regionalized changes, the embryonic development of the axial skeleton, including the rib cage and primordial shell, remains evolutionarily conserved. Comparative embryological studies on shell kinesis have thus focused on the appendicular skeleton.

**Fig. 6:**
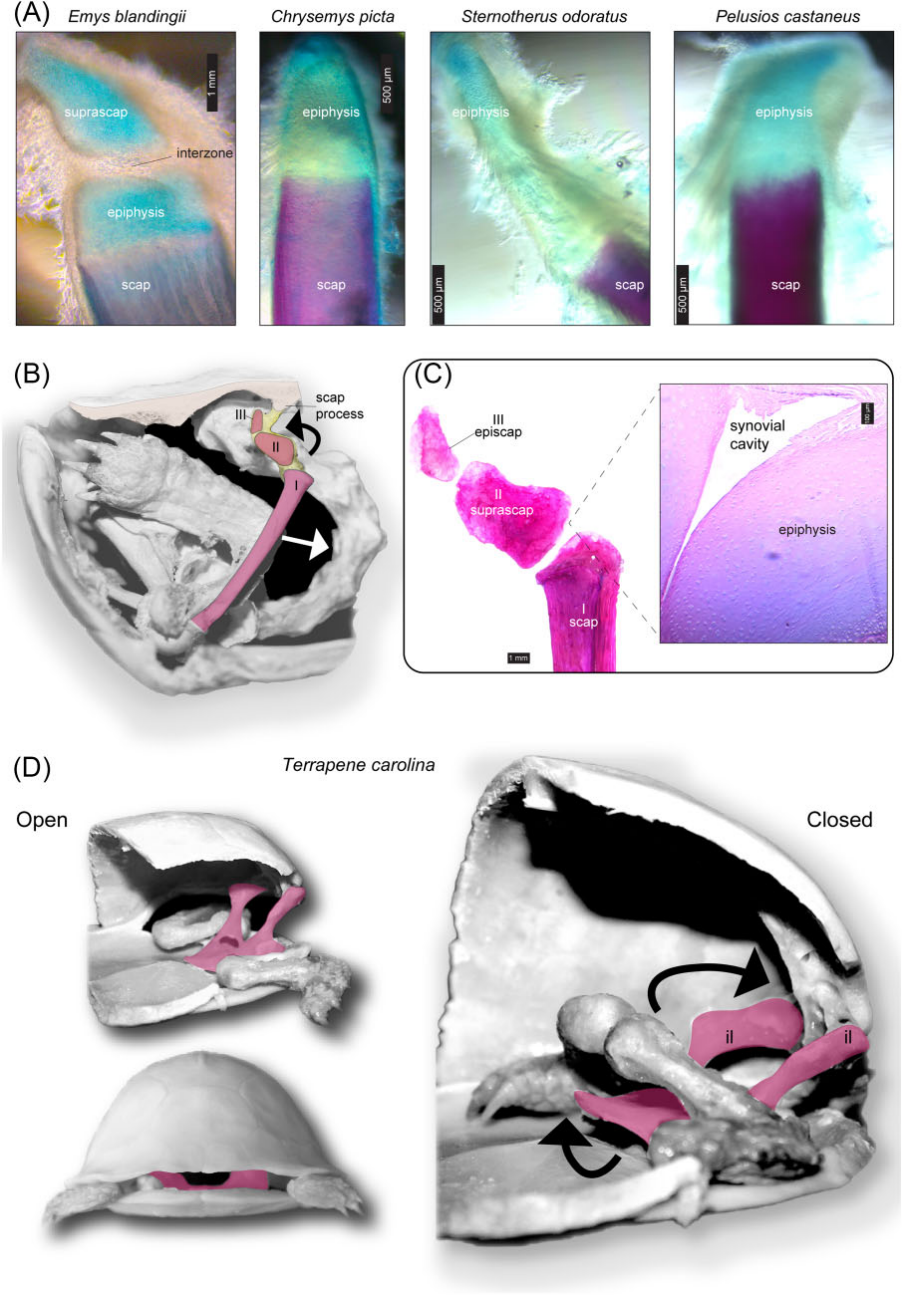
(**A**) The dorsal scapula is modified in some species with shell kinesis, such as *Emys blandingii*, whose hatchlings feature at least one supernumerary bone (e.g., suprascapula) derived from embryonic scapular cartilage (stained in Alcian blue). The scapula is a single continuous unit with a cartilaginous terminus, the epiphysis, that articulates to the visceral surface of the carapace, for example, *Chrysemys picta*. The cartilaginous epiphysis is disproportionately larger in the kinetic-shelled *Sternotherus odoratus*, while kinetic-shelled *Pelusios castaneus* displays a posteriorly oriented epiphysis. Scapulae were dissected from late-term embryos, except *P. castaneus*, by Cordero and Quinteros (2015). (**B**) Withdrawal of the appendicular skeleton and accommodation within the shell cavity is facilitated by the anterior-to-posterior displacement (see arrows) of a tripartite scapula in *Terrapene carolina*: I = scapula; II = suprascapula; III = episcapula. (**C**) Mobility is accomplished via the rotation of a specialized diarthrodial (synovial) joint that separates the suprascapula and scapula. Inset: the synovium in longitudinal section stained in hematoxylin and eosin. (**D**) During shell closure, the ventral pelvic girdle (highlighted in pink) is pulled upwards (see arrows) via a modified ilia (il)–sacral rib articulation (not shown). Skeletal preparations of adult specimens were originally performed in Cordero et al. (2018a).

Bramble mentioned that the embryonic scapula undergoes segmentation in emydid box turtles and recognized that no other vertebrate animal exhibits this condition (Bramble 1974). This observation was validated, in addition to revealing surprising interspecific variation in the timing of skeletal differentiation processes that yielded similar tripartite scapulae in *Terrapene* and *Emys* (Cordero and Quinteros 2015; Cordero et al. 2018a). Other limb girdle modifications also originate in embryos (Fig. 6A). The unusually flexible scapular prong in kinosternids appears to be prepatterned, as nearly half the length of the dorsal scapula remains cartilaginous in hatchlings or late-term embryos of *Sternotherus* and *Kinosternon* (Cordero and Quinteros 2015). This atypical condition may be associated with the capacity for the adult dorsal scapula to stretch to accommodate the head and limbs during shell retraction (Bramble et al. 1984). A similar movement is accomplished via the antero-posterior displacement of the tripartite scapula in emydid box turtles (Fig. 6B and C). Pelvic girdle modifications also occur (see Fig. 6D), though they have not yet been examined in embryos.

**Fig. 7:**
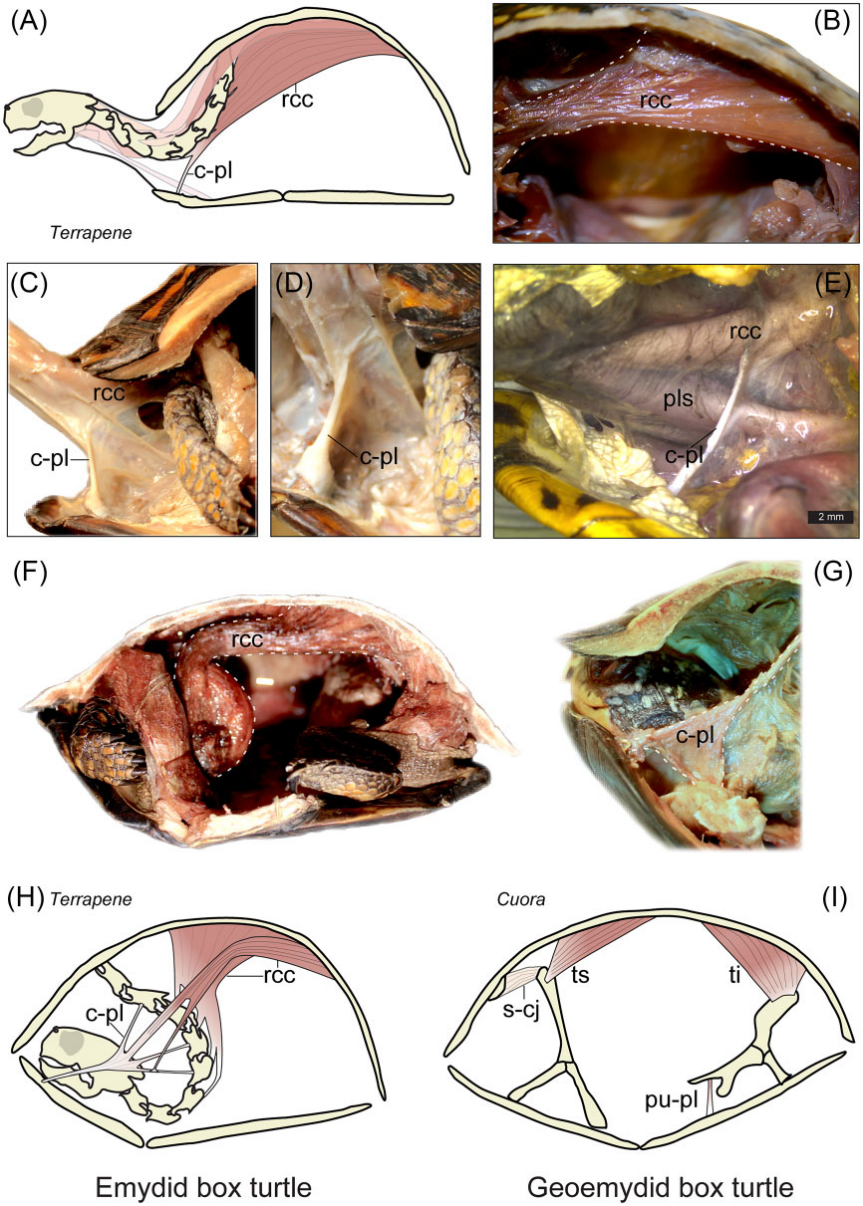
(**A**) The primary neck-retracting muscle, the *retrahens capitis collique* (rcc), has undergone change in some turtles with plastral kinesis, e.g., *Terrapene carolina* (**A**). (**B**) The rcc originates along the dorsal vertebrae (posteriorly), has several insertion sites on the cervical vertebrae that also share a paired ligamentous connection to the visceral surface of the epiplastron, which was first referred to as the cervico-plastral ligament (c-pl in panels **C–E**) by Bramble (1974). (**C**–**D**) The insertion of the c-pl (left side shown) to the epiplastron was confirmed in formalin-fixed specimens of *T. carolina*. (**E**) It was also observed *post mortem* in a freshly dissected road-killed specimen of *Terrapene ornata*, in which the rcc could be clearly differentiated from the plastrosquamosus (pls) that otherwise lacks insertion sites on the cervical vertebrae. During *ex vivo* retraction of the neck in preserved *T. carolina* specimens, the rcc was contracted (panel **F**) and the c-pl exhibited tension as the anterior plastral lobe was pulled upwards (panel **G**). (**H**) Idealized cartoon models of the c-pl as the rcc is contracted in *Terrapene* spp., based on the depiction of Bramble (1974) and observations made herein. (**I**) Asian box turtles (*Cuora* spp.) were hypothesized by Bramble (1974) to feature a shell-closing system wherein the pectoral girdle acts as a lever when the *testoscapularis* (ts) muscle is adducted. Movement of the pectoral girdle is facilitated by the stretching of the scapulo-carapacial joint (s-cj) capsule. (**I**) Bramble (1974) further proposed that the *testoiliacus* (ti) pulls on the posterior plastron via the pubis-plastron ligament (pu-pl) that concomitantly raises the ventral pelvic girdle.

**Fig. 8:**
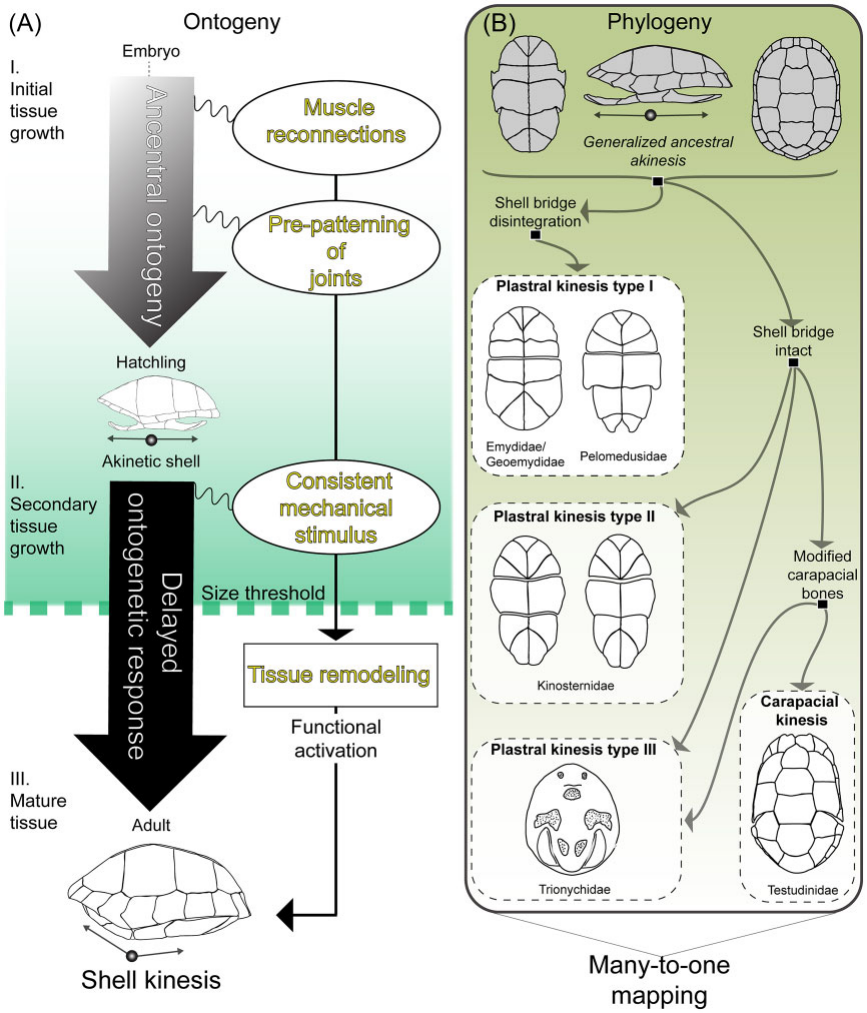
(**A**) Hypothetical model for ontogenetic changes related to the delayed emergence and functional activation of shell kinesis in turtles. (I in panel **A**) During initial tissue growth, the “reconnection” of muscles is probably the first qualitative change (relative to the ancestral ontogeny) observed in the musculoskeletal system. Thereafter, limb girdle joints are pre-patterned in late-term embryos. (II in panel **A**) During post-hatching (secondary) tissue growth, novel muscle connections and mobility of limb girdle components likely generate the consistent mechanical stimulus necessary for bone sutures to undergo remodeling, thus contributing to the functional activation of kinesis via flexion of a mature hinge in adults (III in panel **A**). (**B)** Three forms of plastral kinesis (types I–III), plus one of carapacial kinesis, have arisen independently from multiple akinetic ancestors in turtle phylogeny (see Fig. 2). Considering that different suite of correlated muscle and limb girdle modifications led to shell mobility in unrelated lineages, the evolution of shell kinesis supports the concept of “many-to-one-mapping,” that is, that similar functional outcomes may evolve via different rearrangements of the musculoskeletal system (Wainwright 2007).

Muscle attachment sites are determined together with the establishment of the primordial skeleton (Hall 2015). Functionally relevant evolutionary changes in the structural coupling of bone and muscle (see Fig. 7) may therefore be traced to embryonic stages. In addition to muscle hypertrophy (Cordero et al. 2018a), the convergent origins of shell kinesis depend on muscle topographical shifts in embryos (see “reconnections” in Fig. 8A) that subsequently elicit shell tissue reorganization in juveniles. Such minor muscle reconnections are more likely to be integrated into the highly canalized ontogenetic sequence of turtle embryos and may co-evolve with lineage-specific shell morphologies (Table 1; Fig. 8B). The convergent evolution of shell kinesis has not entirely adhered to predictable ontogenetic trajectories: Different musculoskeletal rearrangements in embryos have yielded structurally and functionally similar hinge phenotypes in adults. Hinges may develop in anatomically homologous shell regions, but their movement depends on the action of different muscle groups, as discussed in the section “Musculosketal evolvability in turtles with shell kinesis”.

**Table 1.** Summary of anatomical alterations associated with the convergent evolution of shell kinesis in modern turtles.

		Key anatomical alterations		
Taxa	Type/location	Principal adductor muscle(s)	Skeletal articulations/accessory structures	Sources
Order Pleurodira				
Pelomedusidae: *Pelusios*	I. Single plastral: hyoplastron–mesoplastron	A derivative of the *diaphragmaticus*, the *levator plastralis*, is the adductor of the anterior lobe	Large but moveable axillary process disarticulated from carapace	Bramble and Hutchison (1981)
Order Cryptodira				
Emydidae: *Emys* and *Terrapene*	I. Single plastral: hyoplastron–hypoplastron	Adduction of anterior plastral lobe via the cervico-plastral ligament of a hypertrophied *retrahens capitis collique*; the *testoiliacus* is the adductor of the posterior plastral lobe	Scapulo-carapacial joint with accessory bones; elastic ligament connecting last dorsal vertebra with first (mobile) sacral vertebra	Bramble (1974)
Geoemydidae: *Cuora* and *Cyclemys*; (*Notochelys*?)	I. Single plastral: hyoplastron–hypoplastron	A hypertrophied *testoscapularis* is the adductor of the anterior plastral lobe, whereas the *testoiliacus* is of the posterior plastral lobe	Dorsal scapula articulates to carapace via synovial capsule with a meniscus; ilial–carapacial ball-and-socket joint with pubis-to-plastron ligament	Bramble (1974)
Kinosternidae: *Kinosternon, Sternotherus*, and *Staurotypus*	II. Single or double plastral: epiplastron–hyoplastron and/or hypoplastron–xiphiplastron	Adduction of anterior plastral lobe via the cervico-plastral ligament of the *retrahens capitis collique*; the *attrahens pelvium* and *testoiliacus* are adductors of the posterior plastral lobe (with an ischio-xiphiplastral ligament in *Kinosternon*)	Extensive dorsal scapula cartilage attached to carapace via a synovial capsule	Bramble et al. (1984)
Trionychidae: *Lyssemys*	III. Single plastral*: entoplastron-hyoplastron	Equivocal; adduction of anterior lobe possibly by cutaneous tension coupled with contraction of the *nucho-epiplastralis* (possibly homologous to the *trapezius*); posteriorly, skin valves close the caudal aperture via contraction of the *flexor caudae superficialis* and via a ligament on the femur	Accessory bones on posterior margin and anterior end of carapace; carapace and plastron extended by skin appendages (valves)	Hasan (1941); George and Shah (1955); Shah (1960)
Testudinae: *Kinixys*	Single carapacial: costals 4–5 and peripherals 7–8	Equivocal; posterior bundles of the *retrahens capitis collique* may contribute to adduction of the carapacial lobe, together with contraction of the *attrahens pelvium*	Slightly mobile thoracic vertebrae 4–5 with reduced fusion to the carapace	Siebenrock (1916); Shah (1960)

* *Lyssemys* spp. also feature changes in the carapace that enhance shell closure.

## Musculosketal evolvability in turtles with shell kinesis

Comparative analyses on adults have mainly focused on the external morphology of shell kinesis (Claude 2006; Angielczyk et al. 2011; McLaughlin and Stayton 2016; Cordero et al. 2019, 2023), though a handful of studies provided detailed descriptions of the cervical and limb girdle musculature (Shah 1960; Bramble 1974; Bramble and Hutchison 1981; Bramble et al. 1984). The latter studies demonstrated that shell kinesis is a multidimensional phenotype with various musculoskeletal features that have not been described in any other vertebrate animal. A comparative anatomical review is thus necessary to discuss hypotheses on how or whether the turtle musculoskeletal system has been predictably transformed during the evolution shell kinesis.

### Diverse muscle “reconnections” underlie functional coupling in the evolution of kinesis

One of the most common muscle modifications in the convergent evolution of shell kinesis concerns the gain of a novel connection of the primary neck-retracting muscle (the *retrahens capitis collique*) to the plastron, which facilitates elevation of the anterior plastral lobe (Table 1; Fig. 7A–H). A total of 5 of the 10 genera with plastral kinesis feature this key connection, though it is otherwise absent in geoemydid box turtles (*Cuora*), softshell turtles (*Lyssemys*), and side-neck turtles (*Pelusios*) (Table 1; Fig 7I). It may therefore be hypothesized that different muscle “reconnections” arose due to the ancestral skeletal state of subclades (Table 1). Crucially, ancestral shell features of a given subclade should not necessarily be viewed as constraints, but rather as factors that bias the direction of evolution. For instance, softshell turtles (*Lyssemys*) with plastral kinesis employ a shell-closing mechanism that relies on shell flexibility afforded by the absence of scutes, as well as dermal extensions of the carapace and plastron that function as flaps that shield the head and limbs (Table 1; Fig. 2C) (Hasan 1941). Because the *retrahens capitis collique* is not connected to the plastron in *Lyssemys* (George and Shah 1955), the stretching of skin and connective tissues has been hypothesized to contribute to plastral lobe elevation (Hasan 1941).

Among hard-shelled turtles, sideneck species (Pleurodira) may be less likely to evolve kinesis because they retract the neck laterally. As a result, contractile forces generated via the adduction of the primary neck-retracting musculature are not directed upward, as opposed to hidden-neck species (Cryptodires) (Fig. 7). Instead, the *diaphragmaticus* in pleurodires (*Pelusios*) is hypothesized to have been subdivided into a novel muscle, the *levator pastralis*, which assists in the elevation of the anterior plastron via a connection to a degraded axillary buttress that functions as a lever (Bramble and Hutchison 1981). Because the inguinal buttress of the shell bridge region remains intact in *Pelusios*, movement of the posterior plastral lobe would require many more modifications to achieve. By contrast, cryptodires that exhibit remodeling of the inguinal buttress do elevate the posterior lobe, in addition to the anterior lobe (Type I plastral kinesis in Fig. 8B). Although the shape of the shell in cryptodire species with Type I plastral kinesis (emydid and geoemydid box turtles) is strikingly similar (McLaughlin and Stayton 2016), correspondingly similar muscle topographical changes are not shared by these species. This key distinction in the muscles that power kinesis in emydid and geoemydid box turtles may be explained by stochasticity, that is, chance events in evolution. In any case, variation in muscle rearrangements, that is, shifts in insertion and origination sites, are not functionally trivial because they directly influence the distribution of forces generated by muscle adduction. Plastral lobe elevation in emydid box turtles primarily relies on the adduction of the *retrahens capitis collique*, which is hypertrophied and features ligamentous extensions with insertion sites on the plastron (*Terrapene*; Fig. 7A–E), which is otherwise absent in akinetic emydid turtles (Scanlon 1982). Bramble (1974) referred to this meshwork of ligamentous bundles as the “cervico-plastral ligament” because it establishes a connection from the cervical vertebrae to the visceral surface of the epiplastron bones. As a result, the cervical vertebrae that are directly affected by the adduction of the *retrahens capitis collique* during neck retraction are functionally and structurally coupled to the anterior plastral lobe, as confirmed by dissection of museum specimens (Fig. 7B–H). In geoemydid box turtles, hypertrophied *testoscapularis* and *testoiliacus* are hypothesized to be the principal adductors of plastral lobe elevation (Fig. 7I). In addition, the origin sites for the *testoscapularis* are shifted posteriorly, which is compensated by a bulging out of adjacent spinal nerves (Bramble 1974). Bramble (1974) also depicted a ligament connecting the pubis and plastron that further assists in the elevation of the posterior plastral lobe in geoemydid box turtles (Fig. 7I). Similar pelvic ligaments or repositioning of the shoulder blade musculature has not been described in emydid box turtles (see Fig. 7G and H).

The location of hinges is more distal to the shell bridge region in kinosternid species that exhibit little or no modification of the axillary or inguinal shell buttresses (Type II kinesis), though these species may also feature a similar cervico-plastral ligament as emydid box turtles (Table 1; Fig. 8B). Thus, Bramble et al. (1984) proposed that the *retrahens capitis collique* is also the main adductor muscle during shell closure in Kinosternidae (Type II kinesis; Table 1, Fig. 8B). Interestingly, the structural correlates of the second (posterior) hinge displayed by most kinosternids include a ligament that connects the ischium of the pelvic girdle to the plastron, as well as a subdivided *attrahens pelvium* that is also connected to the plastron (Bramble et al. 1984). It may be hypothesized that the intensity of muscle-derived forces is augmented and the conditions that favor posterior hinge development are met via a reinforced pelvis–plastron connection, possibly as a result of higher selective pressure for posterior shell closure in the kinosternids (i.e., *Kinosternon*) that frequently utilize terrestrial environments (Wygoda and Chmura 1990). Anatomical correlates of double plastral kinesis in Kinosternidae would suggest that muscle-derived forces, if permitted to be consistently transmitted to skeletal tissue at the proper location, may influence the plastic expression of a functionally relevant phenotype. Although this assumption is supported by experiments on joint development in vertebrate laboratory models (Drachman and Sokoloff 1966; Kahn et al. 2009; Ennos 2011; Eyal et al. 2015; Felsenthal and Zelzer 2017), further functional studies are needed in turtles (Cordero 2020).

### Correlated limb girdle alterations, innovation, and functional enhancement

There are clear tradeoffs related to the rigidity of a shelled skeleton that encases the limb girdles. Nevertheless, the limb girdles are far from static structures in turtles (Walker 1974). For instance, the inability for turtles to perform lateral undulations during locomotion is compensated by rotational excursions of the shoulder and pelvic girdle (Mayerl et al. 2019). This capacity for the limb girdles to move was further refined during the convergent evolution of shell kinesis. Once muscle connectivity, orientation, and subdivision are ontogenetically diverged from the ancestral (akinetic condition) of a taxon, modified limb girdle articulations that enable kinesis continue to transform until they become fully functional in adults (Fig. 8A). Such delayed transformations permit the translation, rotation, and stretching of girdle components during shell closure (Bramble 1974; Bramble and Hutchison 1981; Bramble et al. 1984). The articulation of the shoulder blade (scapula) and visceral surface of the carapace is normally comprised of a ligamentous connection (Fig. 6A), which typically limits the mobility of the shoulder girdle. In the highly specialized box turtles, shoulder girdle movement clears space for the forelimbs to be fully retracted within the shell cavity (Fig. 6B), while possibly providing mechanical leverage that contributes to plastral lobe elevation (Bramble 1974).

During shell closure, evolutionarily novel accessory bones (the suprascapula and episcapula) are set in motion and diverted away from a fossa that lies adjacent to the first thoracic rib of emydid box turtles (Fig. 6B). Movement is facilitated by a synarthrodial joint with a synovium that reduces friction during folding and antero-posterior displacement of the episcapula, suprascapula, and scapula (Fig. 6B and C). Geoemydid box turtles (*Cuora*) lack accessory bones and the dorsal scapula is instead moved via stretching of its ligamentous connection to the carapace (Bramble 1974). The intricacy of this scapulo-carapacial joint is impressive, as it features a meniscus with two synovial cavities (Bramble 1974). Additional anatomical analyses are needed to further clarify these details and ascertain whether they are generalizable to other geoemydid genera with plastral kinesis. As with variation in muscle modifications, geoemydid box turtles employ different skeletal alterations of the limb girdles in comparison to emydid box turtles.

A unique ball-and-socket joint permits anterodorsal rotation of the pelvis in geoemydid box turtles (*Cuora*), whereas the sacrum is projected anteriorly such that it slides over the last thoracic vertebra in emydids (*Terrapene*) (Bramble 1974). Raw anatomical and *ex vivo* examinations validate pelvic rotation in *Terrapene* (see Fig. 6D), yet, the ilio-carapacial joint and sacrum await examination. The pelvic girdle also rotates along the anterodorsal axis to enhance the retraction of the hind limbs in hinge-back tortoises, that is, *Kinixys* (Shah 1960; Cordero et al. 2023). The movement of sacral ribs during pelvic rotation in *Kinixys* should be described and compared to emydid box turtles, as these modifications represent interesting case studies of how the lumbar-to-thoracic transition may evolve without a change in the regional identity of vertebral segments (e.g., mammals; Galis et al. 2014). Such specialized skeletal articulations may be regarded as evolutionarily novel because they have not been described, at least in terms of topography, in other vertebrate groups, but also because they expand the functional limits of the appendicular skeleton. As such, intricate transformations of the limb girdles corroborate the expectation that the development of a shelled body has redirected the evolution of the turtle musculoskeletal system toward a range of evolvable character states (phenotypic space), which is generally not exploited by other vertebrate animals, possibly owing to a lack of selection for the correlated traits that otherwise functionally enhance the shelled body plan of turtles.

## On the predictability of musculoskeletal evolution

Developmental or structural constraints on the vertebrate musculosketal system are not an absolute hinderance to evolution; rather, they may slow down evolutionary rates and thus reduce the frequency of certain phenotypes across vast geological time scales. Along these lines, Raff (1996) stated: “*If turtles didn't exist, we might have predicted that the placement of the shoulder girdle is internally constrained and impossible to change. The mere prevalence and long-term conservation of a particular body plan element does not indicate that it is impossible to make a transition to a different element*.”

### Constraints versus opportunities

**Fig. 9:**
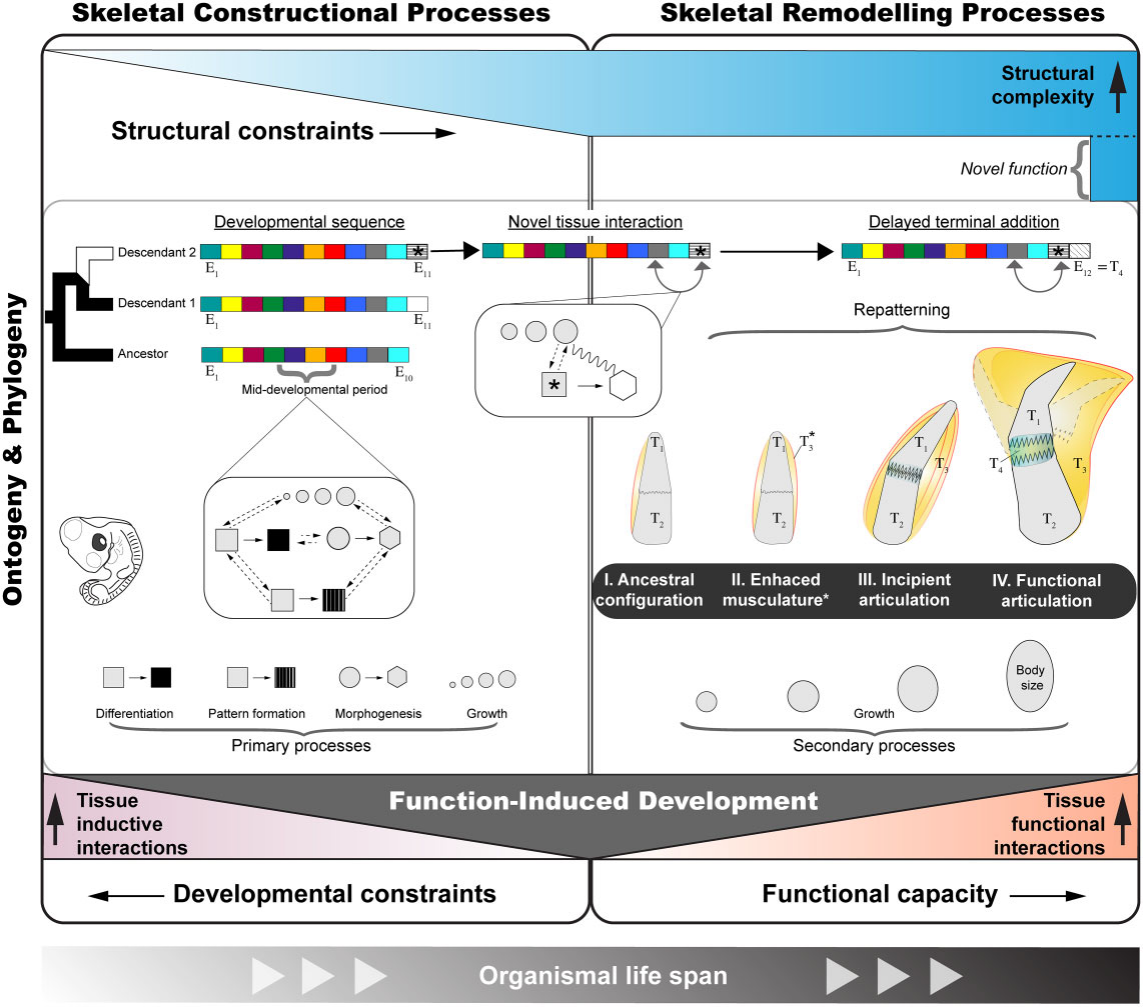
Hypothetical framework for function-induced development of musculoskeletal traits in vertebrate animals. Constructional processes (left panel) of the primordial musculoskeletal system are depicted as a tightly synchronized sequence of developmental events (E_1. . ._). Events situated early in the sequence tend to be highly evolutionarily conserved. Toward the middle of the sequence (mid-developmental period), tissue (inductive) interactions intensify as developing traits become increasingly complex and integrated within the embryo. Evolutionary change in skeletal constructional processes most likely occurs via terminal additions (see E_11_) to the ancestral sequence, thereby bypassing developmental constraints related to the initial events by which the embryonic skeleton arises. Because structural complexity increases as ontogeny ensues, evolutionary change may be further constrained. However, in some special cases (see E_11_ with asterisk), a novel tissue functional interaction may later stimulate skeletal remodeling (right panel). In the right panel, a hypothetical example is given of adjacent skeletal elements (T_1–2_) that have gained additional muscle connections (T_3_) in evolution. As body size increases along with tissue functional interactions, muscle-derived forces may serve as inputs for skeletal tissue to gradually undergo remodeling. The end result of this process is a functional articulation (T_4_) that bisects the skeletal elements and enhances mobility beyond what was feasible in the ancestral configuration, that is, a novel function. This class of skeletal transformation may unfold across embryonic and post-embryonic life stages, such that functional bone–muscle tissue interactions also exert inductive control over organismal development.

The turtle's shell is considered an evolutionary novelty because no other vertebrate clade features similar modifications of the rib cage and shoulder girdle, most of which have persisted since the Late Triassic (Cordero 2017; Lyson and Bever 2020). Still, innovation may beget innovation. Convergent kinetic shells exemplify novel opportunities for an ancient body plan to change in response to clade-specific functional demands. Since the early Paleogene, the turtle musculoskeletal system has likely undergone myriad alterations associated with the complex biomechanical systems that enable shell kinesis. The anatomical overview presented herein does not paint a picture of wholly predictable macroevolutionary change, which contradicts the conjecture of relatively low phenotypic diversity in turtles. Along these lines, vertebrate paleontologist Rainer Zangerl referred to the turtle's shell as an “evolutionary straitjacket” in terms of constrained adaptive diversification, though he recognized that such a notion is overstated (Zangerl 1969). The convergent evolution of shell kinesis is a testament to the latter because, upon close anatomical examination, it is evident that modern turtles have capitalized on new opportunities for the vertebrate musculoskeletal system to evolve. Constraints related to the establishment of the primordial (shelled) skeleton in embryos might have been mitigated by terminal additions to the ancestral sequence of developmental events in turtles (Fig. 9).

Even though turtles share similar developmental constraints, the correlated response to selection for shell kinesis has not played out in a predictable manner. The various means by which the cervical and appendicular muscles were functionally coupled to shell bones is consistent with the “many-to-one-mapping” expectation for the evolution of the vertebrate musculoskeletal system (Alfaro et al. 2005; Wainwright 2007; Muñoz 2019). Similarity in shell morphology alone is not sufficient to predict which muscles may be potentially recruited as integral components of kinetic shells. Still, it may be predicted that kinesis will, with some exceptions, require the enlargement and reconnection of the neck and limb girdle musculature. Enlarged neck muscles may feature ligamentous insertion sites on the cervical vertebrae that are rerouted and linked to the plastron. In the absence of this key structural association, oversized shoulder blade muscles may originate at a greater distance along the vertebral column to produce greater power and use the shoulder blade as a lever during shell closure. Under this configuration, the redirection of pelvic muscle forces via modified ligaments to the plastron may reinforce leverage. A second hinge may even develop if the functional coupling of the pelvis and plastron is fortified by additional pelvic muscle connections. As the functional cooperation of muscle and shell bone is established, the concomitant movement of the pelvic or shoulder girdles within the rigid shell cavity demands the development of highly specialized articulations, none of which are identical in the species studied thus far.

Although all kinetic-shelled species share in common the delayed emergence of at least one kinetic hinge in juveniles, this is likely the end product of a sequence of transformations that generate consistent mechanical stimulus required for sutural degradation (tissue remodeling) during joint differentiation (Fig. 4D). At the cellular and tissue scale, not much interspecific variation is expected during this process. Instead, it is how the requisite mechanical stimulus is generated, which depends on historical contingency, that is, the ancestral phenotypic parameters of a clade (Lewis 2018) (see ``ancestral ontogeny” in Fig. 8A). The timing or threshold for hinge differentiation in post-hatching life is likely determined by species-specific growth rates and intensity of mechanical stimuli experienced by developing skeletal tissue, which is probably set by the overgrowth (hypertrophy) and rearrangement of muscles and ligaments that occurs earlier during embryonic life stages (Table 1; Fig. 8A). Not all muscle connections are amenable to modification, though this has promoted unprecedented phenotypes. This is perhaps best exemplified by the hypothesized exaptation of pulmonary muscles in sideneck turtles, which was likely favored because, during shell closure, it may be the only means by which contractile forces can be transmitted to the plastral lobe while retaining the ancestral pleurodiran mode of lateral neck retraction. Alternatively, the rarity of this phenotype among pleurodires may be the result of strong selection for plastral kinesis in the small-sized and sometimes semi-terrestrial *Pelusios*. It has been hypothesized that selection for shell kinesis is favored in small-bodied species that sometimes inhabit terrestrial environments where predator avoidance is challenging (Green 1988; Cordero et al. 2018b).

The fusion of thoracic vertebrae with neural bones of the carapace is one of the most invariable features of the turtle's shell (Zangerl 1969; Lyson and Bever 2020), yet it was partially reduced to permit carapacial kinesis by somewhat freeing thoracic vertebrae 4–5 from the shell enough to undergo dorsoventral flexion during shell closure (Siebenrock 1916). Based on its low frequency and absence in the fossil record, it may be surmised that carapacial kinesis is less likely to evolve because it would entail exceptional transformations of the thoracic vertebrae and adjacent shell bones. Such structural limitations related to a shelled body were mitigated by skeletal remodeling in hinge-back tortoises (Cordero et al. 2023). At the tissue level, the resorption of shell bones that surround thoracic vertebrae is not a trivial process because it necessitates the activity of osteoclasts (bone-deconstructing cells) that respond to changes in the mechanical stress regime experienced by developing tissue (Bourne 1971; Currey 2002; Cowin 2004; Hall 2015). In fact, it was proposed that during skeletal remodeling a tissue-level response similar to what would be observed during the wound healing process may be employed, albeit at a rather slow pace during juvenile stages (Cordero et al. 2022). Furthermore, hinge differentiation possibly involves adaptive reorganization processes that contribute to the repatterning of ectodermal structures (scutes), as in elephants and crocodiles (Milinkovitch et al. 2013; Martins et al. 2018). Precisely how muscle contractile forces are redistributed sufficiently to trigger tissue plasticity remains largely underappreciated, even though it has long been recognized that such skeletal reaction norms may prime the evolution of adaptive phenotypes (Waddington 1957; Müller 1990; Young and Badyaev 2007; Herring 2011; Wagner 2014).

### Beyond turtles: comparisons to the convergent evolution of cranial kinesis in vertebrates

Multiple forms of cranial kinesis have evolved across the vertebrate tree of life and the adaptive value of this trait is often unequivocal (Frazzetta 1970; Arnold 1988; Gans 1988; Bock 1999; Herrel et al. 2000; Iordanskiĭ 2000; Bout and Zweers 2001; Holliday and Witmer 2008; Mezzasalma et al. 2014; Bailleul et al. 2017; Cost et al. 2020; Ivanović et al. 2022). Similar to turtle shell kinesis, it has been postulated that cranial kinesis is the result of changes in trait correlations, for example, in Archosaurs (Felice et al. 2019). The closure of shell bone sutures is mechanistically similar to the suturing of skull bones in vertebrate animals (Depew et al. 2008; Rice and Rice 2008). Just as in shell sutures (Sarnat and McNabb 1981), incipient skull bone sutures are highly dynamic structures that are subject to growth and resorption, that is, remodeling (Rice and Rice 2008). An advantage of examining turtle ontogeny is that such remodeling processes occur at a relatively low rate that can be documented across the lengthy life span of some species. An interesting prospect to follow up on is whether the differentiation of kinetic cranial joints follows similar skeletal remodeling processes as those described in turtles. Although the development of cranial kinesis has only been studied a handful of times (Wake and Hanken 1982; Tokita 2003), much may be inferred by comparing the representative adult stages of diverse species that independently evolved the trait. This may further elucidate that the skeletal remodeling processes associated with cranial and shell hinge joints, as well as specialized limb girdle joints, might be part of a common strategy to overcome developmental and structural constraints on the evolution of the vertebrate musculoskeletal system.

## Conclusions and broader considerations

The structural and functional covariance of skeletal and muscle tissue has been long recognized in mammals (Wolff 1892; Zelditch et al. 2008; Cornette et al. 2015; Cordero and Berns 2016). Using the laboratory mouse, as well as chick embryos, experimental evidence that muscle contraction is critical to the developmental origins of skeletal articulations and related bones has been available for several decades (Drachman and Sokoloff 1966; O’Rahilly and Gardner 1978; Hall 1986). These studies followed the landmark experiments of Hampé (1959), wherein various reptile-like limb joint phenotypes were induced by interfering with muscle function in developing chicks (Müller 1989; Müller and Streicher 1989). More recently, similar instances of function-induced development were experimentally supported in evo-devo research on fish (Hu and Albertson 2017) and birds (Botelho et al. 2015; Woronowicz et al. 2018). In agreement with predictions from evo-devo theoretical models (Alberch 1985; Alberch and Blanco 1996), skeletal remodeling processes that are presumably activated by muscle function are likely to be observed after the primordial embryonic skeleton is established. As such, early-occurring constructional processes of the primordial skeleton may be regarded as constraints, as these tend to be highly evolutionarily conserved and are assumed to be sensitive to mutations (Galis and Metz 2019; Cordero et al. 2020). As ontogeny progresses, structural complexity should increase, which may further limit changes to the skeleton. Skeletal remodeling processes might be a response to such constraints. To illustrate this point, a hypothetical framework for how function-induced development contributes to the adaptive evolution of the vertebrate musculoskeletal system is depicted in Fig. 9. Under this model, traits that arise via the terminal addition or rearrangement in the sequence of embryonic developmental events may yield altered tissue functional interactions that simultaneously serve as tissue inductive interactions that are necessary for the structural rearrangement and origination of additional skeletal elements (Fig. 9). Such tissue remodeling processes are probably integral to the evolution of novel musculoskeletal traits, because they leverage the malleability of skeletal tissue in response to altered mechanical stress regimes. However, much more experimental evidence is needed to conclusively validate the generality of function-induced development, particularly as it pertains to inferences on the evolutionary process (see discussion in Herring 2011).

The convergent evolution of shell kinesis suggests that constraints might be modulated via ontogenetic transformations that span multiple life stages and various levels of hierarchical biological organization. It should be pointed out that whether phenotypic convergence is a reflection of constraints has and will continue to be a contentious assertion in evolutionary biology (Losos 2011; Agrawal 2017). To this end, the comparative survey presented herein invites further research that explores the rich 210-million-year-old history of turtles and integrates anatomy, functional morphology, and development to further clarify the determinants of convergent evolution via complex changes to the musculoskeletal system. More broadly, the ideas and hypotheses discussed herein may extend to other vertebrate taxa, for example, cranial kinesis, wherein function-induced developmental processes might generate novel opportunities to undergo adaptative change in response to the inherited constraints of a given clade.

## Data Availability

Data will be made available upon request.
